# MSI Analysis in Solid and Liquid Biopsies of Gastroesophageal Adenocarcinoma Patients: A Molecular Approach

**DOI:** 10.3390/ijms22147244

**Published:** 2021-07-06

**Authors:** Elisa Boldrin, Maria Assunta Piano, Rita Alfieri, Marcodomenico Mazza, Loretta Vassallo, Antonio Scapinello, Pierluigi Pilati, Matteo Curtarello

**Affiliations:** 1 Immunology and Molecular Oncology, Veneto Institute of Oncology IOV-IRCCS, Via Gattamelata 64, 35128 Padova, Italy; mariaassunta.piano@iov.veneto.it (M.A.P.); matteo.curtarello@iov.veneto.it (M.C.); 2 Oncological Surgery Unit, Veneto Institute of Oncology IOV-IRCCS, Via dei Carpani 16, 31033 Castelfranco Veneto, Italy; rita.alfieri@iov.veneto.it (R.A.); marcodomenico.mazza@aulss2.veneto.it (M.M.); pierluigi.pilati@iov.veneto.it (P.P.); 3 Pathology Unit, Veneto Institute of Oncology IOV-IRCCS, Via dei Carpani 16, 31033 Castelfranco Veneto, Italy; loretta.vassallo@aulss8.veneto.it (L.V.); antonio.scapinello@iov.veneto.it (A.S.)

**Keywords:** gastroesophageal adenocarcinoma (GEA), microsatellite instability (MSI), cell-free DNA (cfDNA), liquid biopsy

## Abstract

Gastroesophageal adenocarcinoma (GEA) patients with the microsatellite instability (MSI) subtype emerged as optimal candidates for immunotherapy. To date, immunohistochemistry (IHC) is the gold standard for MSI assessment in formalin-fixed paraffin-embedded (FFPE) specimens. However, IHC, although useful for diagnostic typing, cannot be used to analyze cell-free DNA (cfDNA) in liquid biopsy, a tool that could overcome tumor heterogeneity and enable longitudinal monitoring. In order to find an alternative diagnostic method to IHC, we analyzed 86 retrospective GEAs FFPE samples with multiplex PCR. Moreover, to verify the feasibility of MSI detection in liquid biopsy, cfDNA samples of five patients that resulted in having MSI in a prospective cohort of 35 patients were evaluated by multiplex PCR, real-time PCR and droplet digital PCR (ddPCR). Analysis of FFPE showed 100% concordance between multiplex PCR and IHC (Cohen’s Kappa agreement = 1). On the contrary, only ddPCR was able to detect MSI in cfDNAs of T3/T4 GEA patients. In conclusion, data highlight the molecular analysis as an optimal alternative to IHC for the diagnostic typing and suggest that the ddPCR assay can be considered as the most reliable and promising molecular approach to detect MSI in the cfDNA of GEA patients.

## 1. Introduction

Gastric and esophageal adenocarcinomas, characterized by similar genetic and epigenetic molecular alterations, are collectively termed gastroesophageal adenocarcinomas (GEAs). Gastric adenocarcinoma (GAC) is the 5th most common cancer and the 2nd leading cause of cancer-related death [1]. Esophageal adenocarcinoma (EADC) is instead the 8th most frequent cancer, and the 6th principle cause of cancer-related death worldwide [2]. A multimodality approach to treatment, surgery alone or in combination with preoperative chemo- or chemo-radiotherapy, is associated with recent improvements in disease outcomes and survival. In patients with operable disease treated with neoadjuvant chemotherapy before surgery, the 5-year overall survival (OS) is 36%. In advanced or metastatic GAC, 5-year OS is around 5–20% and median OS is under 1 year. However, in EADC, the overall prognosis is worse [3]. To date, no validated biomarkers predictive of the treatment response to therapeutic agents are available in GAC. Exceptions are the overexpression of the human epidermal growth factor receptor-2 (HER2) and programmed death-ligand 1 (PD-L1) for which trastuzumab and pembrolizumab, respectively, have been demonstrated to give clinical benefits [4]. Recently, The Cancer Genome Atlas Research Network proposed a simple and stepwise process to characterize a gastric adenocarcinoma based on its predominant molecular profile. This approach provides prognostic information and suggests a potential benefit from targeted therapy. It is possible to distinguish four GAC subtypes based on genomic characterization: Epstein–Barr virus-positive (EBV; 9%), microsatellite instability (MSI; 22%), chromosomal instability (CIN; 50%) and genomic stable tumors (GS; 20%) [5].

MSI GAC is characterized by a deficiency of at least one protein of the DNA mismatch repair (MMR) system. Moreover, this subtype is frequently characterized by a high mutational burden (14.6–60.9 mutations/Mb) [6,7] with frequent mutations in *PIK3CA*, *ERBB3*, *ERBB2* and *EGFR* [5]. Recent studies have shown that the MSI status predicts the clinical benefit of immunotherapy with PD-1/PD-L1 inhibitors leading to the approval of pembrolizumab for unresectable or metastatic MSI solid tumors, including GEAs, following progression on prior therapies [8]. The exact mechanism of the more efficient response to the immunotherapy of MSI patients is unknown; in any case, it seems that tumors with a high mutational burden (>10 mutations/Mb) express neoantigens that could render them more susceptible to an immune checkpoint blockade [9]. For this reason, the high mutational burden of MSI GAC could explain its good response to treatment.

Currently, MSI testing for immunotherapy decision-making is commonly performed on tissue biopsies using immunohistochemistry (IHC) that is based on the assessment of the MMR proteins’ expression level. However, about 5–11% of MSI GAC that show a normal MMR protein staining and localization could have dysfunctional proteins due to missense mutations, leading to the erroneous exclusion of these patients from immunotherapy [10]. 

Furthermore, tissue biopsies have several limitations since they could not represent the whole tumor status, especially in highly heterogeneous tumors such as gastroesophageal cancer [11], are invasive and make the longitudinal monitoring of the disease status impossible. Thus, the development of new and reliable diagnostic tools transposable also to liquid biopsy is needed. 

Indeed, currently it is recognized that cell-free DNA (cfDNA) gives more comprehensive information regarding tumor burden compared to tissue biopsy by overcoming the challenge of intratumoral heterogeneity [12,13,14]. cfDNA analysis has emerged also as a promising tool to improve the management of GEA patients [12,14,15,16,17,18,19,20,21,22,23].

Taking into account the possibility for MSI patients to have a benefit from immunotherapy, the application of sensitive molecular approaches to improve MSI detection could recover those patients who are currently excluded from a correct therapeutic approach.

In this study, aimed to find a valid alternative to currently used IHC typing, we tested different molecular approaches to analyze MSI status in formalin-fixed paraffin-embedded (FFPE) tissues and in cfDNA of GEA patients. 

## 2. Results

### 2.1. Clinicopathologic Characteristics of Patients

For this study, in order to test the validity of the MSI molecular analysis to implement the MSI GEA diagnostic typing, we retrospectively selected 86 archival FFPE samples from GEA patients; moreover, we collected 35 prospective GEA patients to test the feasibility of applying the MSI molecular analysis to cfDNA isolated from plasma.

Clinicopathologic characteristics of the retrospective and prospective cohorts are shown in Table 1. The median age was higher in the retrospective than in the prospective cohort (76 vs. 68, respectively). The male/female ratio and the distribution of patients in relation to the tumor site were comparable. Locally advanced GEAs were more represented in the prospective cohort (62.8% vs. 44%).

### 2.2. IHC in FFPE Samples of the Retrospective and Prospective Cohorts

Retrospective and prospective cohort patients have been stratified into four different GEA subtypes according to Gonzales R.S. et al. [24]. Stratification was based on the IHC evaluation of MMR proteins expression (MLH1, MSH2, MSH6 and PMS2), p53 alteration and Epstein–Barr virus early RNA (EBER) antigen presence characterizing the MSI, CIN and EBV subtypes, respectively.

In the retrospective cohort, 15 patients (17%) had MSI resulting from MMR protein deficiency, 28 patients (33%) had alterations in p53 revealing a CIN profile and 3 patients (3%) expressed EBER antigen and have been classified as EBV-positive. Forty patients (47%) expressing normal levels of MMR proteins, without alterations in p53 and negative for the EBER antigen, were classified as GS (Table 1).

In the prospective cohort, 5 patients (14%) showed a deficiency in MMR proteins and were classified as MSI, 11 patients (32%) had alterations in p53 revealing a CIN profile and no patients expressed the EBER antigen. Nineteen patients (54%) were classified as GS (Table 1).

**Figure 1:**
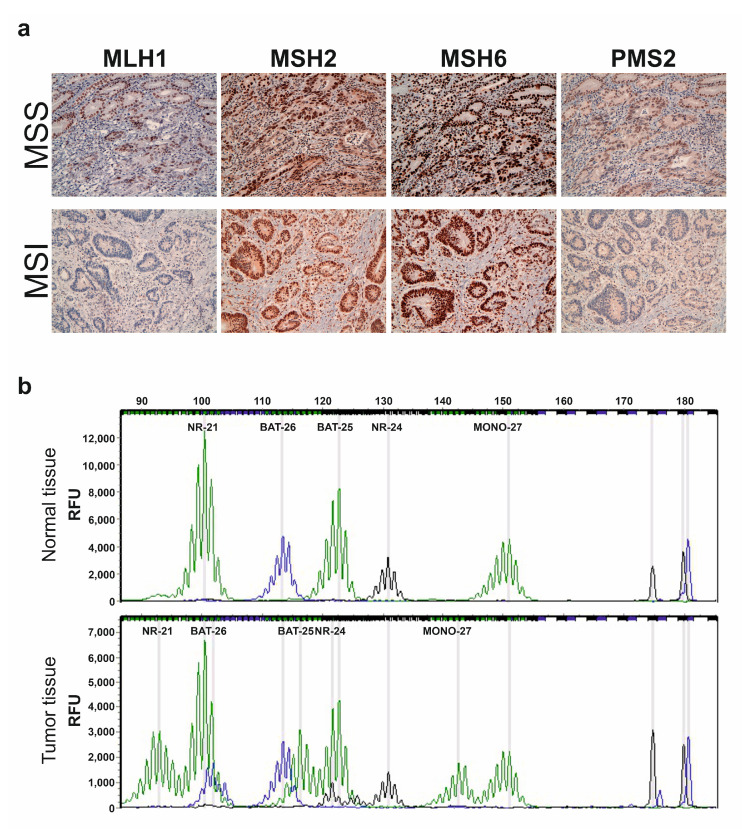
Representative IHC staining for MMR proteins and electropherograms of multiplex PCR analysis using the MSI Analysis System Version 1.2 kit. (**a**) MLH1, MSH2, MSH6 and PSM2 expression in FFPE samples of a MSS and a MSI patient by IHC (original magnification 20×). The MSS patient shows normal expression of all MMR proteins. Deficiency in MLH1 and PSM2 proteins is observed in the MSI patient; (**b**) Multiplex PCR electropherograms show profiles of 5 quasimonomorphic microsatellites (NR-21, BAT-26, BAT-25, NR-24 and MONO-27) in the normal and tumor tissue of the same patient. The tumor tissue shows additional peaks that are absent in the normal tissue of all microsatellites analyzed, revealing an MSI profile.

In both cohorts, the MSI phenotype was mainly characterized by the absence of MLH1 and/or PMS2 expression (data not shown). Figure 1a shows an example of a patient with MLH1 and PMS2 deficiency, compared with a microsatellite stable (MSS) patient that had normal expression of all four MMR proteins.

### 2.3. Validation of the MSI Molecular Assay in FFPE Samples of the Retrospective Cohort

We used the MSI Analysis System Version 1.2 kit, a commercial molecular assay based on multiplex PCR and subsequent capillary electrophoresis that analyzes five quasimonomorphic microsatellites (BAT-25, BAT-26, NR-21, NR-24 and MONO-27), to find an alternative technique to IHC for diagnostic purposes.

To verify the performance of this kit on the FFPE samples, DNA from tumor and normal FFPE specimens of the 86 retrospective patients was analyzed and results were compared with the diagnostic IHC typing. All the 15 patients, which were classified MSI according to IHC, also resulted in having MSI with the molecular analysis. In particular, 14 patients had at least four unstable loci (i.e., high microsatellite instability, MSI-H), while 1 patient had only one unstable locus (i.e., low microsatellite instability, MSI-L). The remaining 71 patients, which were classified as MSS with the IHC, were also confirmed as MSS by the molecular analysis. These results revealed a 100% concordance between the IHC and the multiplex PCR assay (Cohen’s Kappa agreement = 1).

Figure 1b reports an example of an MSI profile in a FFPE tumor sample compared to its normal tissue, analyzed by means of the MSI Analysis System Version 1.2 kit.

### 2.4. MSI Molecular Assay in the cfDNA of the Prospective Cohort

Once the comparable performance of the MSI Analysis System Version 1.2 kit was verified against the gold standard diagnostic IHC, we proceeded to transpose this molecular technique into liquid biopsy in order to detect MSI in the cfDNA of the prospective cohort patients.

**Figure 2:**
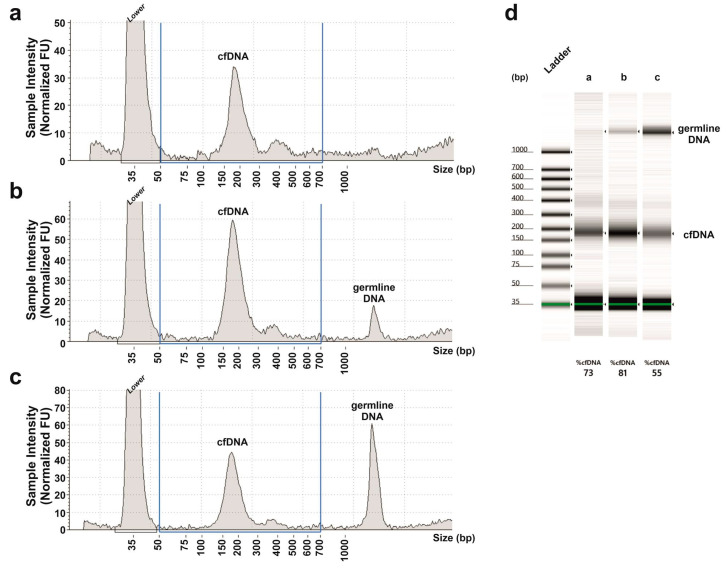
Electropherograms and gel-like image of cfDNA samples analyzed with Agilent TapeStation 2200. The fragments around 35 bps, between 150 and 200 bps and >1000 bps represent the lower marker, the cfDNA and the germline DNA, respectively. Profile of a cfDNA (**a**) without germline DNA contamination, (**b**) with minimal and (**c**) with high contamination; (**d**) electrophoretic runs of the cfDNA samples of panels (**a**–**c**). The percentage of cfDNA is reported below each lane.

All five patients with an MSI status at IHC typing also resulted in having MSI by means of the molecular analysis in FFPE samples. The instability had been found in at least four loci, revealing an MSI-H profile for all five patients. For these patients, cfDNAs obtained just before surgery have been analyzed. Unfortunately, in all patients, MSI was undetectable in cfDNA. To assess if the detection failure was attributed to contamination with germline DNA that could compete with cfDNA in the multiplex PCR reaction, we checked the quality of cfDNAs with Agilent Tape Station 2200. Fragments of 150–200 bps represent the typical pattern of cfDNAs [25] and were found in all our cfDNA samples (Figure 2). Superior-size fragments of >1000 bps indicate contamination with germline DNA. Only cfDNA samples without or with minimal contamination were selected for our experiments (Figure 2a,b).

To further investigate the reason for detection failure, we tested the performance of this kit by analyzing a series of dilutions constituted by a mix of constant tumor DNA amount isolated from the tumor MSI FFPE specimen and crescent amounts of normal (germline) DNA isolated from the matched normal FFPE tissue. The total DNA amount for PCR amplification was 30 ng and the dilutions of tumor DNA/normal DNA were: 1:2, 1:4, 1:8, 1:16 and 1:32. Undiluted tumor DNA and normal DNA were also analyzed as controls and resulted in MSI and MSS, respectively. The kit detected the presence of MSI up to the 1:8 dilution, corresponding to 12.5% of tumor DNA (Table 2). This suggests that the MSI Analysis System Version 1.2 kit is not suitable for MSI analysis in cfDNA.

In order to find a more sensitive molecular technique to detect MSI in cfDNA, we tested the performance of the Easy-PGX ready MSI kit (Diatech Pharmacogenetics) and of the Bio-Rad MSI droplet digital PCR (ddPCR) test (Bio-Rad). The Easy-PGX ready MSI kit is based on eight different real-time PCR reactions analyzing eight quasimonomorphic microsatellites (BAT-25, BAT-26, NR-21, NR-22, NR-24, NR-27, CAT-25 and MONO-27), while ddPCR analyzes the same microsatellite loci investigated by the multiplex PCR assay.

Additionally, for these two kits, a series of tumor DNA/normal DNA dilutions was evaluated, as previously described for the multiplex PCR MSI kit. A total of 50 ng of DNA amount/assay was used.

The Easy-PGX ready MSI kit detected the presence of MSI up to a 1:16 dilution, corresponding to a 6.25% tumor DNA amount, highlighting a low sensitivity, which makes it unsuitable for MSI cfDNA analysis (Table 2); ddPCR still detected MSI up to a 1:512 tumor DNA/normal DNA dilution, corresponding to a 0.2% tumor DNA content (Table 2). At this dilution, instability in three out of five microsatellites was still detectable (data not shown). According to the manufacturer’s instructions of ddPCR, the sample was considered to have MSI when ≥2 altered microsatellites were found.

Based on these encouraging results, we decided to exclude the real-time PCR assay and to use ddPCR to analyze the cfDNAs and the time-matched FFPE DNA of the five MSI prospective patients (GP06, GP24, GP26, GP29 and GP39). For these analyses we started with cfDNA extracted from the minimal volume (1 mL) of plasma and, in the case of undetectable MSI, we used cfDNA from the whole available amount of plasma (3–5 mL).

The FFPE specimens time-matched with cfDNAs of the five prospective patients resulted in all having MSI with ddPCR, showing a 100% concordance with IHC.

Two patients (GP29 and GP39) resulted in being heterozygous at the genomic level in BAT-25 and MONO-27, respectively. Interpretation of the results in heterozygous loci was very difficult and could lead to the identification of false positives so, for this reason, the analysis for these patients was limited to the other four homozygous loci.

**Figure 3:**
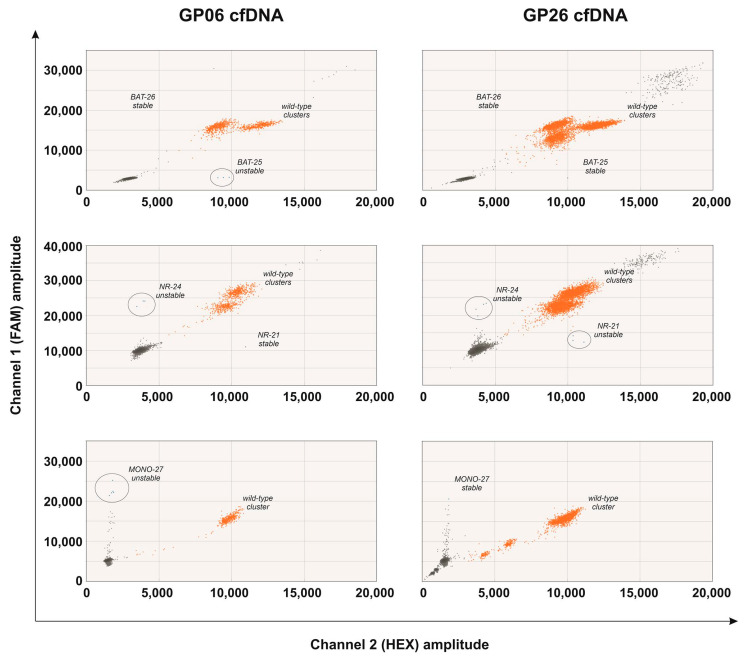
Two-dimensional plot of droplet fluorescence for the three ddPCR MSI assays (BAT-25 and BAT-26; NR-21 and NR-24 and MONO-27) performed in the cfDNA of patients GP06 and GP26. Wild-type molecules are in the orange clusters, and the microsatellite unstable molecules are in the blue clusters. The individual target is labeled on each plot. Circles identify loci with at least three positive droplets (unstable loci).

**Figure 4:**
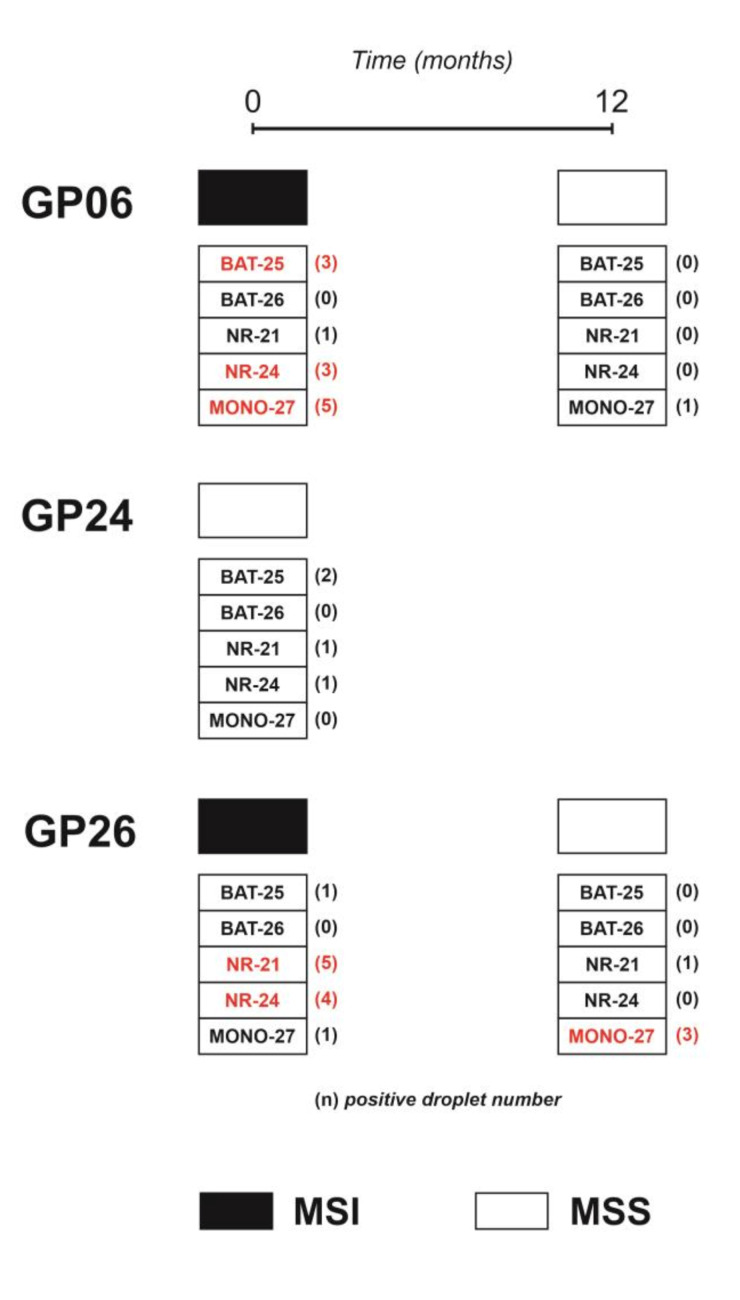
MSI status in cfDNA samples of GP06, GP24 and GP26 prospective patients. Black and white rectangles indicate MSI or MSS status, respectively. Unstable microsatellites are shown in red and the number of positive droplets is indicated in brackets.

At the time of the first blood sampling (first point cfDNA), performed after neoadjuvant chemotherapy and immediately before surgery, patient GP06 presented a gastric adenocarcinoma (ypT4aN3a). ddPCR showed the presence of MSI in 5/5 microsatellites in the tumor DNA from the FFPE sample and in 3/5 microsatellites (BAT-25, NR-24 and MONO-27) in the cfDNA (Figure 3), revealing a MSI profile in both FFPE and cfDNA (Figure 4). MSI was detectable in the cfDNA extracted from the minimum volume of plasma required for extraction (1 mL). At 12 months after surgery, the patient was still disease-free and resulted in being MSS in the cfDNA (second point) extracted from the maximum plasma volume (5 mL) (Figure 4).

GP26, a patient with a locally advanced gastric adenocarcinoma (pT3N1) who did not receive neoadjuvant therapy, presented alterations in all five analyzed microsatellites in the FFPE sample and only in two loci (NR-21 and NR-24) in the first point cfDNA collected immediately before surgery (Figure 3 and Figure 4). MSI was detectable in the cfDNA extracted from 5 mL of plasma, while it was undetectable in the cfDNA extracted from 1 mL. In this patient, the second point cfDNA, collected at 12 months after surgery, resulted in being MSS at the maximum volume of plasma (5 mL) (Figure 4).

Patient GP24, a ypT2N0 GEA, resulted in having MSI in FFPE with 4/5 altered microsatellites. In the cfDNA sample, extracted from 5 mL of plasma collected after neoadjuvant therapy and before surgery, ddPCR detected at least one positive droplet in 3/5 microsatellites but, as the cut-off of three positive droplets in at least two microsatellites was not reached, the sample was considered to be MSS.

Patient GP29, a ypT1bN0 GEA had 4/4 altered microsatellites in FFPE, resulting in having MSI. MSI was undetectable in the time-matched cfDNA extracted from the maximum amount of plasma available (3 mL) after neoadjuvant therapy. Finally, patient GP39, a pT2N2 GEA, resulted in having MSI in FFPE with 4/4 altered microsatellites, but was MSS in the cfDNA extracted from the maximum amount of plasma available (4.7 mL).

## 3. Discussion

Gastroesophageal adenocarcinoma is still a poor-prognosis cancer. However, recent promising findings on the clinical benefit of pembrolizumab for MSI GEAs provided the rational for the improvement of MSI detection techniques in FFPE specimens and plasma. Indeed, IHC, which still represent the gold standard for diagnostic typing, is time-consuming and costly and not feasible for longitudinal monitoring of patients. On the other hand, the PCR-based analysis in FFPE samples has demonstrated that it is an optimal alternative to IHC in colorectal cancer [26]. Moreover, the molecular MSI detection permits one to identify also the cases with a normal expression of dysfunctional MMR proteins, explaining the discordance observed between IHC and molecular tests in the literature [10,27].

Our result showed that the MSI molecular analysis could be used for GEA typing. Indeed, the MSI Analysis System Version 1.2 kit had a 100% concordance with IHC, suggesting that this kit could replace IHC staining for MMR proteins in the diagnostic typing of MSI patients. Due to its competitive cost, this method can be a convenient alternative to IHC for MSI detection. Moreover, this technique enables the analysis of all the microsatellites in only one well due to its multiplex approach, thus saving time, sample material and costs. Unfortunately, multiplex PCR is not sensitive enough to detect MSI in cfDNA. The real-time PCR-based kit, despite an optimal concordance with IHC, has also proven to be not sensitive enough for cfDNA analysis.

The failure to find MSI in liquid biopsy with multiplex and real-time PCR-based approaches could be explained by considering that cfDNA is a mixture of DNA of different origin (normal cells and tumor cells) and it could be characterized by a low amount of tumor DNA [28].

Only ddPCR, for its nature that consists in the partitioning of the sample into droplets, permits one to dilute away the normal DNA background maximizing the chance of rare alterations detection. Indeed, ddPCR has been widely used in liquid biopsy studies due to its high sensitivity in detecting copy number variations (CNVs) and single nucleotide variations (SNVs) [29]. Only recently, ddPCR assay was used to detect MSI in cfDNA samples of patients with advanced colorectal and endometrial cancers [30].

In our study, we found that the ddPCR MSI assay was able to detect MSI up to 0.2% of tumor DNA. In two GEA patients (GP06 and GP26), hospitalized for the surgical procedure with a T3/T4 tumor, ddPCR successfully detected MSI in cfDNA, while in the other three patients with a T1/T2 tumor (GP24, GP29 and GP39) MSI was undetectable by ddPCR, also using 3–5 mL of plasma for cfDNA extraction.

Taken together, our data suggest that the MSI multiplex PCR-based technique could be an optimal alternative to IHC in GEA diagnostic typing, while MSI detection in liquid biopsy requires the highly sensitive ddPCR technique. To date, few data are available concerning the detection of MSI in GEAs. To our knowledge, only one study investigated and successfully detected MSI in six patients through the direct sequencing of microsatellite regions [14].

The main limit of this study was the restricted number of MSI patients in our prospective cohort, which depended on the fact that the patients were selected only from our Institute from 2019 to 2020 and that the MSI group was not the main abundant GEA subtype (22%).

Nevertheless, although further studies in a larger cohort are needed, our preliminary data suggest that MSI detection with ddPCR is possible in the cfDNA of T3/T4 GEA tumors. However, MSI detection in patients with smaller tumor mass (T1/T2) seems to be more challenging.

In our opinion, MSI-ddPCR is a good promising new tool with the potential to be used in liquid biopsy. This gives the opportunity, in the future, to carry out longitudinal studies in GEA patients to follow the natural tumor history.

## 4. Materials and Methods

### 4.1. Patients

We retrospectively selected 86 gastroesophageal adenocarcinoma (GEA) patients who referred to the Pathology Unit of the Veneto Institute of Oncology (IOV-IRCCS) between 2016 and 2019.

Inclusion criteria were: (i) a histological diagnosis of GEA (all stages); (ii) the availability of a formalin-fixed paraffin-embedded (FFPE) tumor block of a diagnostic biopsy or surgery resection; (iii) the immunohistochemistry (IHC) typing for DNA mismatch repair (MMR) proteins, p53 and the Epstein–Barr virus early RNA (EBER) antigen.

We enrolled 35 prospective GEA patients who referred to the Oncological Surgery Unit of IOV-IRCCS between 2019 and 2020. A “GP” internal code and a progressive number were assigned to prospective patients samples.

Inclusion criteria were the same as those of the retrospective cohort. Exclusion criteria were the concurrent diagnosis of a synchronous or metachronous tumor within 5 years.

One blood sample was collected for this prospective cohort at the time of diagnosis or at the revaluation after neoadjuvant chemotherapy (first point). A blood sample was collected also at the 12th month follow-up after surgery (second point) for some patients. Time-matched FFPE specimens were also analyzed to evaluate concordance between the first point cell-free DNA (cfDNA) and the tumor DNA.

The study was carried out according to the Code of Ethics of the World Medical Association (Declaration of Helsinki and its later amendments) and had the approval of the Comitato Etico per la Sperimentazione Clinica (CESC) of the Veneto Institute of Oncology (cod. number CESC IOV: 2019/72). All subjects involved in the study gave their written informed consent in accordance with the Helsinki Declaration.

### 4.2. Immunohistochemistry

According to the IHC current diagnostic typing protocol for GEAs, all patients were tested for MMR proteins (MLH1, MSH2, MSH6 and PMS2) overexpression, p53 alterations and for the presence of the EBER antigen. The assays were carried out in 4 μm of FFPE tissue sections using the Ventana Benchmark ULTRA platform (Roche, Monza, Italy), following the manufacturer’s instructions. Each staining pattern was evaluated by a senior pathologist, according to the ASCO/CAP protocol for immunohistochemistry interpretation.

The MMR status in GEA specimens was performed with the VENTANA MMR IHC Panel (Roche) that includes four primary antibodies that target proteins: anti-MLH1 (M1) mouse monoclonal, anti-PMS2 (A16-4) mouse monoclonal, anti-MSH2 (G219-1129) mouse monoclonal and anti-MSH6 (SP93) rabbit monoclonal antibodies. In the MMR analysis, the presence of any nuclear staining within the tumor, even if patchy or weak, or the absolute absence represents the “no loss” and the “loss” of targeted MMR proteins expression, respectively. Hence, carcinoma was considered with microsatellite instability (MSI) if the nuclear staining was absent for at least one protein.

The p53 and Epstein–Barr virus (EBV) status in the GEA sections were assessed with the anti-p53 (Bp53-11) primary antibody assay and in situ hybridization Inform EBER Probe (Roche), respectively. p53 IHC evaluation was considered as a positive phenotype when at least 70% of tumor cells disclosed strong nuclear immunostaining. The p53 alteration defined the chromosomal instability (CIN) subtype.

For the EBER analysis, samples were considered positive if nuclear expression of EBER in almost all the neoplastic cells was observed. The presence of the EBER antigen defined the EBV subtype.

Normal levels of MMR proteins and the absence of p53 alterations and of EBER antigen defined GS patients.

### 4.3. DNA Extraction

FFPE tumor DNA was isolated from eight consecutive 10 µm thick sections using the QIAamp Mini Kit (Qiagen, Milan, Italy) according to the manufacturer’s instructions. The DNA quantity and quality was assessed using the NanoDrop 1000 spectrophotometer (Thermo Fisher Scientific, Monza, Italy). A neoplastic component ≥70% was considered adequate for tumor-DNA analysis; where necessary, samples were enriched by manual macrodissection.

Peripheral blood samples were collected in cell-free DNA BCT tubes (Streck, La Vista, NE, USA). Plasma was isolated from corpuscular components of the blood by centrifugation at 2.000× *g*, subsequently centrifuged a second time at 16.000× *g* to remove cellular debris and then stored at −80 °C, until cfDNA extraction. The cfDNA was extracted using the Maxwell RSC ccfDNA Plasma Kit (Promega, Milan, Italy) from 1 mL (minimum volume) of plasma. Another extraction from all the available plasma of the patient (3–5 mL; maximum volume) was performed in the event that MSI was undetectable. The quantity of cfDNA was assessed with the Qubit dsDNA HS Assay kit (Thermo Fisher Scientific, Monza, Italy). The amount of cfDNA ranged between 6 and 30 ng/mL plasma. The quality of cfDNA samples was evaluated by means of the Agilent TapeStation 2200 using the cell-free DNA screen tape assay kit (Agilent Technologies, Milan, Italy). Examples of cfDNA quality are shown in Figure 2. cfDNA samples with a consistent contamination of germline DNA (Figure 2c) were excluded from molecular analyses.

### 4.4. MSI Molecular Analysis

To assess MSI status, we used three commercial kits: MSI Analysis System Version 1.2 (Promega, Milan, Italy), Easy-PGX ready MSI (Diatech Pharmacogenetics, Jesi, Italy) and the Bio-Rad MSI droplet digital PCR (ddPCR) test (Bio-Rad, Milan, Italy). According to the revised Bethesda guidelines, the sample was considered to be MSS if all microsatellites present no length changes, MSI low (MSI-L) if 1 microsatellite was altered and MSI high (MSI-H) with ≥2 altered microsatellites [31]. MSI molecular analyses were performed in a blind manner in the retrospective cohort. Reproducibility was assessed by reanalyzing a few randomly selected samples.

#### 4.4.1. Multiplex PCR

The MSI Analysis System Version 1.2 is a fluorescent multiplex PCR-based assay, which includes a panel of 5 quasimonomorphic microsatellites (BAT-25, BAT-26, NR-21, NR-24 and MONO-27) characterized by mononucleotide repeats and 2 pentanucleotide repeats microsatellite markers (Penta C and Penta D). PCR amplification was performed in 10 μL according to the manufacturer’s instructions using 30 ng of FFPE DNA or 0.8–4 ng of cfDNA. PCR products were separated by capillary electrophoresis using an ABI PRISM 3730xl Genetic Analyzer and analyzed with GeneMapper software v.5 (Applied Biosystems, Monza, Italy). The five mononucleotide markers were selected for their high sensitivity and specificity to determine MSI, while the two pentanucleotide markers were used to detect potential sample mix ups or contamination. MSI was defined as a change in the length of a microsatellite allele, due to the insertion or deletion of the repeat unit. The use of quasimonomorphic microsatellites characterized by mononucleotide repeats eliminates the need to compare the tumor profile with the profile of a reference normal tissue. In any case, the analysis was repeated by adding the matched normal tissue as a reference, in the event of an ambiguous interpretation of the tumor tissue profile.

#### 4.4.2. Real-Time PCR

The Easy-PGX ready MSI kit is a real-time PCR-based assay that analyzes 8 quasimonomorphic and mononucleotide repeats microsatellite markers (BAT-25, BAT-26, NR-21, NR-22, NR-24, NR-27, CAT-25 and MONO-27) in eight different reactions. Each real-time PCR reaction was performed in 25 μL, according to the manufacturer’s instructions, using 50 ng of FFPE DNA or 0.6–3 ng of cfDNA. PCR products were evaluated by using Easy-PGX qPCR Instrument 96 and EasyPGX Analysis Software v.3.0.0 (Diatech Pharmacogenetics, Jesi, Italy). In this assay, each marker is amplified and the determination of the MSI sample profile takes place through a step of denaturation/hybridization with specific dyes. The kit reveals MSI as the presence of a change in the length of a microsatellite allele due to deletions of the repeat unit; the sequence archived in the NCBI database is used as a reference.

#### 4.4.3. ddPCR

The Bio-Rad MSI ddPCR test is based on the highly sensitive molecular approach of ddPCR and utilizes competitive probe drop-off assays with two probes, labeled with FAM or HEX fluorophores, competing for the same target sequence. The analyzed microsatellites were: BAT-25, BAT-26, NR-21, NR-24 and MONO-27 and were analyzed in three different assays (assay 1: BAT-25 and BAT-26; assay 2: NR-21 and NR-24; assay 3: MONO-27). PCR amplification was performed into two replicates of 20 μL each, according to the manufacturer’s instructions, with 50 ng of FFPE DNA or with 1.6–8 ng of cfDNA (1 mL of plasma)/assay or 1.6–12 ng of cfDNA (5 mL of plasma)/assay. PCR products were analyzed by using the QX200 droplet reader and QuantaSoft^TM^ Pro Analysis Software (Bio-rad, Milan, Italy). Both probes bind if the microsatellite has no changes in length (due to deletions of the repeat unit) resulting in a double positive droplet (FAM+HEX+); if the microsatellite has length changes, one of the probes is out-competed, resulting in a single positive droplet (FAM+ or HEX+). According to the manufacturer’s instructions, each microsatellite is considered unstable if at least three single positive droplets are detected across the merge of two replicates. To set the threshold for the correct droplet clusters classification (positive or negative), we performed a ddPCR analysis of FFPE DNAs from MSI patients and cfDNAs extracted from the plasma of healthy donors as controls. The rejection criterium for the exclusion of a reaction from the subsequent analysis was a low number of droplets measured (<10.000 per 20 μL of PCR). The individual droplet (individual partition) volume was 1 nL. To design, perform and report ddPCR experiments Digital MIQE Guidelines were followed when applicable [32].

### 4.5. Statistics

To calculate the concordance between the MSI molecular analysis, performed with MSI Analysis System Version 1.2, and IHC, we applied the Cohen’s Kappa agreement index. The sample size (86 patients) used for the concordance analysis between the molecular test and IHC was sufficient to reach a minimum acceptable Cohen’s Kappa of 0.7 (one-sided), setting type I error to 0.001 and type II error to 0.10 (90% power).

## Figures and Tables

**Table 1:** Clinicopathologic characteristics of gastroesophageal adenocarcinoma (GEA) patients, retrospective and prospective cohorts.

	Retrospective Cohort	Prospective Cohort
Patients	Total	Total
	*N* (%) 86 (100)	*N* (%) 35 (100)
**Age**		
Median (Q1;Q3)	76 (68;82)	68 (57;74)
(Range)	(44–97)	(40–96)
**Gender**		
Male	52 (60%)	21 (60%)
Female	34 (40%)	14 (40%)
**Tumor Site**		
Cardia	14 (16%)	7 (20%)
Fundus	14 (16%)	7 (20%)
Body	31 (37%)	10 (29%)
Antrum	27 (31%)	11 (31%)
**pTNM Stage**		
I/II	48 (56%)	11 (31.5%)
III/IV	38 (44%)	22 (62.8%)
unknown		2 (5.7%)
**IHC Typing**		
MSI	15 (17%)	5 (14%)
CIN	28 (33%)	11 (32%)
EBV+	3 (3%)	0 (0%)
GS	40 (47%)	19 (54%)

Q1: first quartile; Q3: third quartile; MSI: microsatellites instability; CIN: chromosomal instability; EBV: Epstein–Barr virus; GS: genomic stable.

**Table 2:** Comparison of MSI detectability in the three commercial kit at the different tumor DNA/Normal DNA dilutions.

	MSI Analysis System Version 1.2	EasyPGX Ready MSI	MSI ddPCR Assay
Tumor DNA:Normal DNA Dilutions		MSI Status	
1:0	●	●	●
1:2	●	●	●
1:4	●	●	●
1:8	●	●	●
1:16	○	●	●
1:32	○	○	●
1:64	ND	ND	●
1:128	ND	ND	●
1:256	ND	ND	●
1:512	ND	ND	●
1:1024	ND	ND	○

MSI: microsatellites instability; ● MSI Detectable; ○ MSI Undetectable; ND: not done.

## Data Availability

The data presented in this study are available on request from the corresponding author. The data are not publicly available due to privacy restrictions.

## Abbreviations

CIN	Chromosomal instability
CNV	Copy number variation
cfDNA	Cell-free DNA
ddPCR	Droplet digital PCR
EADC	Esophageal adenocarcinoma
EBV	Epstein–Barr virus
FFPE	Formalin fixed paraffin embedded
GAC	Gastric adenocarcinoma
GEA	Gastroesophageal adenocarcinoma
GS	Genomic stable
HER2	Human epidermal growth factor receptor-2
IHC	Immunohistochemistry
MMR	Mismatch Repair
MSI	Microsatellite instability
MSI-H	Microsatellite instability high
MSI-L	Microsatellite instability low
MSS	Microsatellite stable
OS	Overall survival
PD-1	Programmed cell death protein 1
PD-L1	Programmed death-ligand 1
SNV	Single nucleotide variation

## References

[1] Bray F., Ferlay J., Soerjomataram I., Siegel R.L., Torre L.A., Jemal A. (2018). Global cancer statistics 2018: GLOBOCAN estimates of incidence and mortality worldwide for 36 cancers in 185 countries. CA Cancer J. Clin..

[2] Domper Arnal M.J., Ferrández Arenas Á., Lanas Arbeloa Á. (2015). Esophageal cancer: Risk factors, screening and endoscopic treatment in Western and Eastern countries. World J. Gastroenterol..

[3] Choi A.H., Kim J., Chao J. (2015). Perioperative chemotherapy for resectable gastric cancer: MAGIC and beyond. World J. Gastroenterol..

[4] Bang Y.-J., Van Cutsem E., Feyereislova A., Chung H., Shen L., Sawaki A., Lordick F., Ohtsu A., Omuro Y., Satoh T. (2010). Trastuzumab in combination with chemotherapy versus chemotherapy alone for treatment of HER2-positive advanced gastric or gastro-oesophageal junction cancer (ToGA): A phase 3, open-label, randomised controlled trial. Lancet.

[5] (2014). Cancer Genome Atlas Research Network. Comprehensive molecular characterization of gastric adenocarcinoma. Nature.

[6] Dulak A.M., Stojanov P., Peng S., Lawrence M.S., Fox C., Stewart C., Bandla S., Imamura Y., Schumacher S.E., Shefler E. (2013). Exome and whole-genome sequencing of esophageal adenocarcinoma identifies recurrent driver events and mutational complexity. Nat. Genet..

[7] Kim S.T., Cristescu R., Bass A.J., Kim K.-M., Odegaard J.I., Kim K., Liu X.Q., Sher X., Jung H., Lee M. (2018). Comprehensive molecular characterization of clinical responses to PD-1 inhibition in metastatic gastric cancer. Nat. Med..

[8] Marcus L., Lemery S.J., Keegan P., Pazdur R. (2019). FDA Approval Summary: Pembrolizumab for the Treatment of Microsatellite Instability-High Solid Tumors. Clin. Cancer Res..

[9] Schumacher T.N., Schreiber R.D. (2015). Neoantigens in cancer immunotherapy. Science.

[10] Dudley J.C., Lin M.-T., Le D.T., Eshleman J.R. (2016). Microsatellite Instability as a Biomarker for PD-1 Blockade. Clin. Cancer Res..

[11] Battaglin F., Naseem M., Puccini A., Lenz H.-J. (2018). Molecular biomarkers in gastro-esophageal cancer: Recent developments, current trends and future directions. Cancer Cell Int..

[12] Gao Y., Zhang K., Xi H., Cai A., Wu X., Cui J., Li J., Qiao Z., Wei B., Chen L. (2017). Diagnostic and prognostic value of circulating tumor DNA in gastric cancer: A meta-analysis. Oncotarget.

[13] Pectasides E., Stachler M.D., Derks S., Liu Y., Maron S., Islam M., Alpert L., Kwak H., Kindler H., Polite B. (2018). Genomic Heterogeneity as a Barrier to Precision Medicine in Gastroesophageal Adenocarcinoma. Cancer Discov..

[14] Maron S.B., Chase L.M., Lomnicki S., Kochanny S., Moore K.L., Joshi S.S., Landron S., Johnson J., Kiedrowski L.A., Nagy R.J. (2019). Circulating Tumor DNA Sequencing Analysis of Gastroesophageal Adenocarcinoma. Clin. Cancer Res..

[15] Hamakawa T., Kukita Y., Kurokawa Y., Miyazaki Y., Takahashi T., Yamasaki M., Miyata H., Nakajima K., Taniguchi K., Takiguchi S. (2015). Monitoring gastric cancer progression with circulating tumour DNA. Br. J. Cancer.

[16] Kinugasa H., Nouso K., Tanaka T., Miyahara K., Morimoto Y., Dohi C., Matsubara T., Okada H., Yamamoto K. (2015). Droplet digital PCR measurement of HER2 in patients with gastric cancer. Br. J. Cancer.

[17] Fang W.-L., Lan Y.-T., Huang K.-H., Liu C.-A., Hung Y.-P., Lin C.-H., Jhang F.-Y., Chang S.-C., Chen M.-H., Chao Y. (2016). Clinical significance of circulating plasma DNA in gastric cancer. Int. J. Cancer.

[18] Normando S.R.C., Delgado P.D.O., Rodrigues A.K.S.B., David Filho W.J., Fonseca F.L.A., Cruz F.J.S.M., Del Giglio A. (2018). Circulating free plasma tumor DNA in patients with advanced gastric cancer receiving systemic chemotherapy. BMC Clin. Pathol..

[19] Shoda K., Ichikawa D., Fujita Y., Masuda K., Hiramoto H., Hamada J., Arita T., Konishi H., Komatsu S., Shiozaki A. (2017). Monitoring the HER2 copy number status in circulating tumor DNA by droplet digital PCR in patients with gastric cancer. Gastric Cancer.

[20] Kato S., Okamura R., Baumgartner J.M., Patel H., Leichman L., Kelly K., Sicklick J.K., Fanta P.T., Lippman S.M., Kurzrock R. (2018). Analysis of Circulating Tumor DNA and Clinical Correlates in Patients with Esophageal, Gastroesophageal Junction, and Gastric Adenocarcinoma. Clin. Cancer Res..

[21] Wang Y., Zhao C., Chang L., Jia R., Liu R., Zhang Y., Gao X., Li J., Chen R., Xia X. (2019). Circulating tumor DNA analyses predict progressive disease and indicate trastuzumab-resistant mechanism in advanced gastric cancer. EBioMedicine.

[22] Wang D.-S., Liu Z.-X., Lu Y.-X., Bao H., Wu X., Zeng Z.-L., Liu Z., Zhao Q., He C.-Y., Lu J.-H. (2019). Liquid biopsies to track trastuzumab resistance in metastatic HER2-positive gastric cancer. Gut.

[23] Chen Z., Zhang C., Zhang M., Li B., Niu Y., Chen L., Yang J., Lu S., Gao J., Shen L. (2019). Chromosomal instability of circulating tumor DNA reflect therapeutic responses in advanced gastric cancer. Cell Death Dis..

[24] Gonzalez R.S., Messing S., Tu X., McMahon L.A., Whitney-Miller C.L. (2016). Immunohistochemistry as a surrogate for molecular subtyping of gastric adenocarcinoma. Hum. Pathol..

[25] Pös O., Biró O., Szemes T., Nagy B. (2018). Circulating cell-free nucleic acids: Characteristics and applications. Eur. J. Hum. Genet..

[26] Tieng F.Y.F., Abu N., Lee L.-H., Ab Mutalib N.-S. (2021). Microsatellite Instability in Colorectal Cancer Liquid Biopsy—Current Updates on Its Potential in Non-Invasive Detection, Prognosis and as a Predictive Marker. Diagnostics.

[27] Hechtman J.F., Rana S., Middha S., Stadler Z.K., Latham A., Benayed R., Soslow R., Ladanyi M., Yaeger R., Zehir A. (2020). Retained mismatch repair protein expression occurs in approximately 6% of microsatellite instability-high cancers and is associated with missense mutations in mismatch repair genes. Mod. Pathol..

[28] Corcoran R.B., Chabner B.A. (2018). Application of Cell-free DNA Analysis to Cancer Treatment. N. Engl. J. Med..

[29] Pantel K., Alix-Panabières C. (2019). Liquid biopsy and minimal residual disease—Latest advances and implications for cure. Nat. Rev. Clin. Oncol..

[30] Silveira A.B., Bidard F.-C., Kasperek A., Melaabi S., Tanguy M.-L., Rodrigues M., Bataillon G., Cabel L., Buecher B., Pierga J.-Y. (2020). High-Accuracy Determination of Microsatellite Instability Compatible with Liquid Biopsies. Clin. Chem..

[31] Umar A., Boland C.R., Terdiman J.P., Syngal S., De La Chapelle A., Rüschoff J., Fishel R., Lindor N.M., Burgart L.J., Hamelin R. (2004). Revised Bethesda Guidelines for Hereditary Nonpolyposis Colorectal Cancer (Lynch Syndrome) and Microsatellite Instability. J. Natl. Cancer Inst..

[32] Huggett J.F., Foy C.A., Benes V., Emslie K., Garson J.A., Haynes R., Hellemans J., Kubista M., Mueller R.D., Nolan T. (2013). The Digital MIQE Guidelines: Minimum Information for Publication of Quantitative Digital PCR Experiments. Clin. Chem..

